# Effect of Cu on the Microstructure and Mechanical Properties of a Low-Carbon Martensitic Stainless Steel

**DOI:** 10.3390/ma15248849

**Published:** 2022-12-11

**Authors:** Jun Ma, Yuanyuan Song, Haichang Jiang, Lijian Rong

**Affiliations:** 1 CAS Key Laboratory of Nuclear Materials and Safety Assessment, Institute of Metal Research, Chinese Academy of Sciences, Shenyang 110016, China; 2 School of Materials Science and Engineering, University of Science and Technology of China, Shenyang 110016, China

**Keywords:** low carbon, martensitic, stainless steel, reversed austenite, Cu-rich precipitates, mechanical properties

## Abstract

Reversed austenite is of vital importance in low-carbon martensitic stainless steel because it improves impact toughness. However, a proper amount of reversed austenite is obtained by tempering at a critical temperature, which reduces the strength of the steel. Therefore, how to improve strength–toughness matching is an important problem. Copper (Cu) is an effective strengthening element in steels. However, there is little in-depth discussion on the role of Cu on the microstructure and mechanical properties of low-carbon martensite steel. In this work, the effect of different Cu content on the reversed austenite formation, tensile strength, and impact toughness of a low-carbon martensitic stainless steel (0Cr13Ni4Mo) was systematically investigated through use of a transmission electron microscope (TEM), transmission Kikuchi diffraction (TKD), atom probe tomography (APT), and other characterization methods and mechanical property tests. The results showed that the addition of Cu decreased the phase transition temperatures of martensite and austenite and increased the volume fraction of the reversed austenite. APT results indicated that Cu-rich clusters first formed with alloying elements such as ferrum (Fe) and nickel (Ni) and then grew to be precipitates through rejection of the alloying elements. The Ni atoms diffused towards the interface between the precipitates and the martensite matrix, which provided heterogeneous nucleation sites for the reversed austenite. Cu precipitations strengthened tensile strength during tempering. However, it generated temper brittleness in the steel at a tempering temperature of 450 °C, resulting in the impact energy of the 3Cu-steel being only 7 J. A good combination with higher tensile strength (863 MPa) and ductility (192 J) was obtained when tempering at 600 °C in the presence of Cu-rich precipitates and a sufficient volume fraction of the reversed austenite. The results provide guidance for the design of steels with reversed austenite and Cu and promote the development of high-strength and high-toughness steels.

## 1. Introduction

Low-carbon martensitic stainless steels are widely used in the nuclear industry [1,2], petrochemical fields, aerospace [3,4], and other industries [5,6] where the combination of strength, toughness, corrosion resistance, and weldability are required [7]. In these steels, reversed austenite is a vitally important phase that transforms into martensite in the process of steel deformation and which can effectively increase the ductility and plasticity of steel, that is, transformation-induced plasticity (TRIP) [8,9,10,11]. Studies have shown that when the volume fraction of reversed austenite reaches 5–20%, a substantial TRIP effect is exhibited [12,13,14]. Reversed austenite can often be obtained by conventional quenching and tempering heat treatment [15,16,17]. However, tempering heat treatment always results in dislocation recovery, which decreases the strength of the steel. Thus, this work aims to answer the question of how to further improve the strength of steels while maintaining good impact toughness.

Copper (Cu), as an effective strengthening element, has often attracted many researchers’ attentions [3,18,19]. The solid solubility of Cu in ferrite (α-Fe) is very low and it is easy to form Cu-rich particles in steel, resulting in a precipitation strengthening effect [20,21,22,23]. This has been widely studied and applied in high-strength steels, such as precipitation-hardened martensite steel, high-strength low-alloy steel (HSLA), and other steels [12,13,14,18,24,25]. However, some studies have found that Cu-rich precipitates often reduce toughness or plasticity while improving strength. For example, when HSLA-100 alloy steel is tempered at 450 °C, although the yield strength is as high as 1168 MPa, impact energy at −20 °C is only 19 J [26]. Therefore, the method of using Cu precipitation strengthening and introducing reversed austenite into the martensite matrix to improve plasticity and toughness provides a better idea for improving the comprehensive properties of materials. Recently, some researchers [27,28] added Cu to 7Ni steel and quenching–partitioning–tempering (Q-P-T) steel to make use of the strengthening effect of Cu-rich precipitates and the toughening effect of reversed austenite to achieve a good strength–toughness match. However, there is little in-depth discussion on the effect of Cu addition on low-carbon martensite stainless steels.

Therefore, this study concentrates on the microstructural characteristics and mechanical properties of the 0Cr13Ni4Mo low-carbon martensitic stainless steel with Cu addition. The influence mechanism of Cu on reversed austenite and mechanical properties was investigated using X-ray diffractions (XRD), a scanning electron microscope (SEM), transmission Kikuchi diffraction (TKD), a transmission electron microscope (TEM), atom probe tomography (APT), and tensile and impact tests. The aim of this work is to explore a method for optimizing the mechanical properties of low-carbon martensite stainless steels. It is believed that the results will provide guidance for the design of steels with reversed austenite and Cu and promote the development of high-strength and high-toughness steels.

## 2. Materials and Methods

The three alloys with different Cu content used in this study were prepared by vacuum melting and their chemical compositions are presented in Table 1. According to Cu content, these three steels are named as 0Cu-steel, 1Cu-steel, and 3Cu-steel, respectively. The three experimental steels were cast into 25 kg ingots and homogenized at 1100 °C for 2 h. These ingots were then hot-forged into cross sections of 70 mm × 35 mm bars, homogenized at 1100 °C for 2.5 h, and hot-rolled into 12 mm thick plates. The test samples were cut from the hot-rolled plates using a wire electrical discharge machining (EDM) machine, homogenized at 1050 °C for 2 h, and then quenched via water cooling. Finally, the samples were tempered at 350 –700 °C for 2 h and then air-cooled.

The phase transformation points of the three steels were measured using an L78RITA-type thermal expansion phase change meter produced by LINSEIS, Germany. The samples were processed into cylindrical rods with a size of Φ3 mm × 10 mm, and the surfaces were guaranteed to be smooth and flat. The heating rate of the samples was 0.5 °C/s, the holding time was 10 min, and the cooling rate was 20 °C/s. Mechanical properties were determined by room-temperature tensile and impact tests. The tensile tests used sheets with a cross-section of 4 mm × 3 mm and were carried out at a strain rate of 10^−3^ s^−1^ at room temperature according to GB/T 228.1-2010 on an INSTRON5982 universal testing system produced by Instron, Britain. The gauge for tensile samples was 20 mm along the hot-rolled direction and yield strength was determined with the 0.2% offset plastic strain method. The size of the impact samples was 10 mm × 10 mm × 55 mm, and the V-notch was perpendicular to the hot-rolled direction. The impact tests were carried out at room temperature according to GB/T 229-2020 on a SANS-ZBC2452-C instrumented impact machine produced by SANS, China. The impact fracture observations were performed using SEM. Sheets with a thickness of 0.5 mm were prepared using a wire EDM machine and mechanically ground to a thickness of 50μm. These foils were then subjected to twin-jet electropolishing in an electrolyte (10% perchloric acid alcohol solution) at −20 °C and 20 V to prepare TEM samples. TEM and scanning transmission electron microscopy (STEM) analyses were carried out on a Talos F200X.

Phase analysis was carried out at a scanning rate of 2 °/min using Cu K_α_ radiation in the 2θ range of 40–102° using a SmartLab multifunctional X-ray diffractometer (XRD) produced by Rigaku, Japan. The volume fraction of reversed austenite was measured by XRD. XRD samples with a size of 10 × 10 × 10 mm^3^ were first carefully mechanically polished and then electropolished with 10% perchloric acid alcohol solution to remove the stress layer. TKD was performed on a ZEISS Gemini SEM 460 Field Emission Scanning Electron Microscope. The step size for TKD scans was 10–20 nm. The method of preparation for TKD samples was the same as for TEM samples.

The APT experiments were performed using the LEAP 5000XR produced by CAMECA, France. For APT samples, cubes with a cross section of 0.5 mm × 0.5 mm were prepared from 3Cu-steel and were then subjected to electropolishing. Rough electropolishing was performed with 25 vol.% perchloric acid in an acetic acid solution using 10 to 20 V direct current (DC) voltage and fine electropolishing was then performed with 4 vol.% perchloric acid in 96 vol.% ethylene glycol butyl ether at 5 to 10 V DC voltage. The needle-shaped samples were analyzed using the laser mode at a low temperature of 50 K and under a high vacuum of ~10^−10^ torr. The detection rate was about 1% and the pulse energy of the laser was 40 pJ. The 3-D reconstruction and analysis were performed using IVAS 3.8.2.

## 3. Results and Discussion

### 3.1. Phase Transition Temperatures

**Figure 1:**
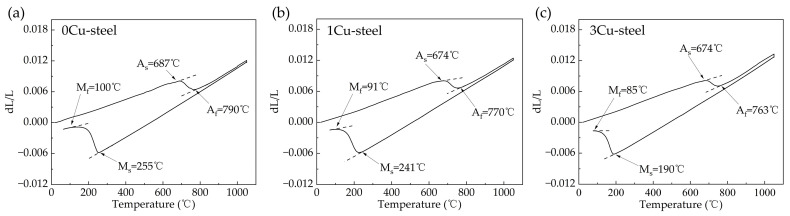
Thermal expansion curves of the three steels with different Cu content: (**a**) 0Cu, (**b**) 1 wt.% Cu, and (**c**) 3 wt.% Cu.

The transformation temperatures of the three steels were determined from the dilatometric curves shown in Figure 1. The detail values calculated by the tangent method are listed in Table 2. From the results, it can be seen that the start temperature of austenite formation (A_s_) and the final temperature of austenite formation (A_f_) in steels with Cu addition are both slightly lower than 0Cu-steel. The start temperature of martensitic transition (M_s_) and the final temperature of martensitic transition (M_f_) decrease significantly with increasing Cu content. This is particularly evident for the steel with 3 wt.% Cu addition, where M_s_ is only 190 °C, which is 65 °C lower than that of 0Cu-steel. This result is roughly consistent with the conclusion that was drawn by Jiao Z.B. et al., who reported that the austenite-to-ferrite transition temperature of Cu/Ni steel decreased by about 46 °C compared to that of Ni steel due to Cu addition [19].

### 3.2. Mechanical Properties

**Figure 2:**
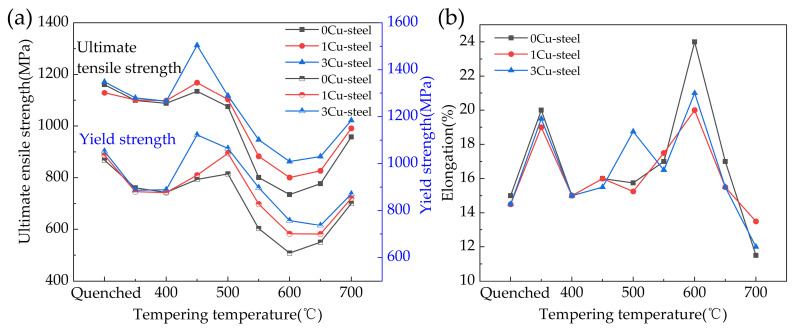
Effect of quenching and tempering temperatures on the mechanical properties of 0Cr13Ni4Mo steels with different Cu content: (**a**) ultimate tensile strength and yield strength; (**b**) elongation.

The tensile properties of the three steels with different Cu content as a function of the quenching and tempering temperature are shown in Figure 2a,b. It can be seen that all three steels showed a certain degree of temper softening. The strength of the three steels was lowest after tempering at 600 °C. Comparing the three curves, the strength of 3Cu-steel is generally higher than that of 1Cu-steel and 0Cu-steel. The ultimate tensile strength and yield strength of the 3Cu-steel both reach very high values at 450 °C: 1313 MPa and 1123 MPa, respectively. This indicates that Cu addition helps to improve the strength of 0Cr13Ni4Mo steel. From the curves displayed in Figure 2b, we can see that the changes in elongation for the three steels in relation to temperature are generally similar. At 600 °C, the 0Cu-steel exhibits better ductility than the 1Cu-steel and 3Cu-steel, reaching 24%.

**Figure 3:**
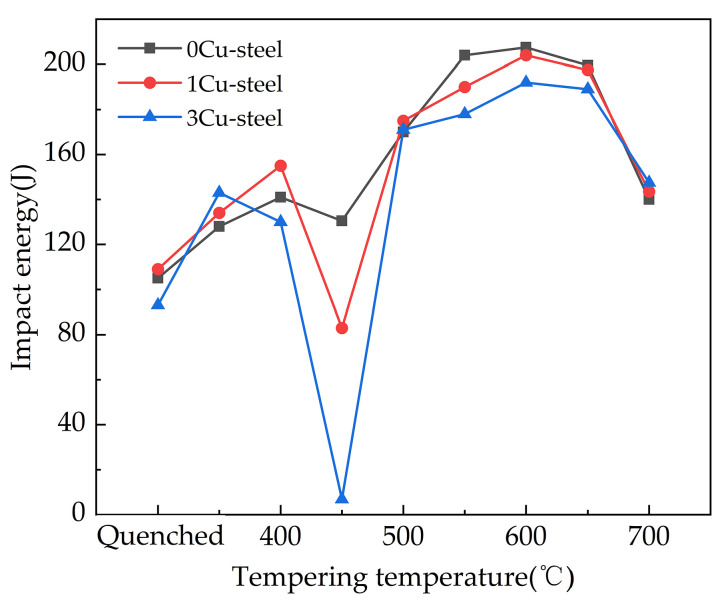
Quenching and tempering temperature dependence for the Charpy impact energy of the 0Cr13Ni4Mo steels with different Cu content.

The impact energy of the three steels as a function of the quenching and tempering temperatures is shown in Figure 3. It can be found that, with the increase in tempering temperatures (except 450 °C), impact energy shows a trend of first increasing and then decreasing, reaching a peak at 600 °C. The peaks for steels with 0Cu, 1 wt.% Cu, and 3 wt.% Cu are 208 J, 204 J, and 192 J, respectively. It is worth noting that Cu addition dramatically reduced the impact energy of steels tempered at 450 °C. It can be seen that the impact energy of 3Cu-steel is reduced to 7 J, which is 124 J lower than that of 0Cu-steel. This suggests that Cu greatly increases temper brittleness at 450 °C. In summary, 3Cu-steel exhibits a satisfactory comprehensive mechanical property after tempering at 600 °C, with an ultimate tensile strength of 863 MPa, a yield strength of 758 MPa, elongation of 21%, and impact energy of 192 J.

**Figure 4:**
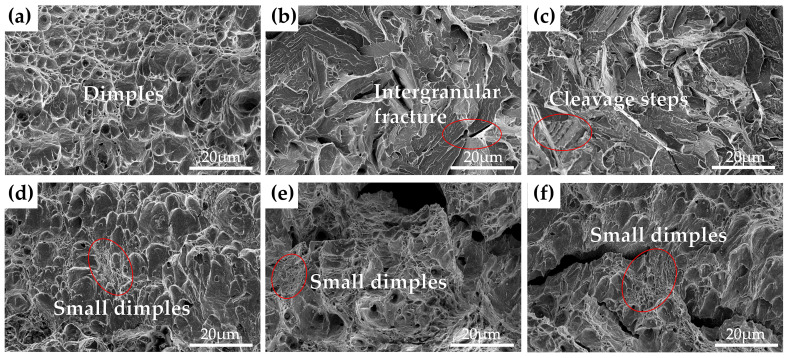
The impact fracture morphologies of (**a**) 0Cu-steel tempered at 450 °C, (**b**) 1Cu-steel tempered at 450 °C, (**c**) 3Cu-steel tempered at 450 °C, (**d**) 0Cu-steel tempered at 600 °C, (**e**) 1Cu-steel at tempered 600 °C, and (**f**) 3Cu-steel tempered at 600 °C.

**Figure 5:**
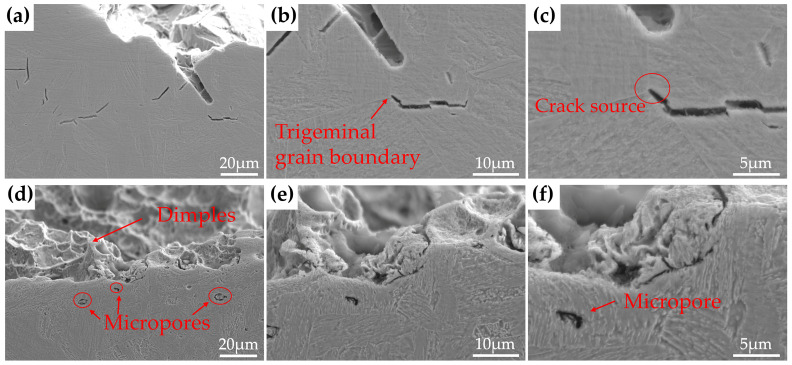
SEM images of different magnification for the cross-sectional areas adjacent to the impact fracture surfaces of 3Cu-steel tempered at different temperatures: (**a**–**c**) 450 °C; (**d**–**f**) 600 °C.

The impact fracture morphologies of the three steels with different Cu content tempered at 450 °C and 600 °C for 2 h are given in Figure 4. It can be observed from Figure 4a that the 0Cu-steel after tempering at 450 °C shows a micro-void coalescence fracture mode with substantial dimples, which are the typical morphology characterization of a ductile fracture; the 1Cu-steel and 3Cu-steel tempered at 450 °C exhibit an opposite mode, which is a typical brittle cleavage fracture, as shown in Figure 4b,c. There are intergranular cracks, cleavage facets, and a lot of cleavage river patterns in the fracture surfaces of the two samples. Meanwhile, the cleavage steps are also detected in Figure 4c. As for the three steels tempered at 600 °C, the surfaces of the impact fracture show a ductile fracture mode with bimodal dimples. SEM images of different magnification for the cross-sectional areas adjacent to the impact fracture surfaces for 3Cu-steel tempered at 450 °C and 600 °C are displayed in Figure 5. It can be clearly observed that the crack source of the 3Cu-steel tempered at 450 °C is mainly located at the grain boundary and the martensitic lath boundaries. From the samples of 3Cu-steel tempered at 600 °C, obvious micropores can be observed, which is a sign of ductile fracture. This indicates that temper brittleness can disappear after increasing the tempering temperature.

### 3.3. Microstructures

#### 3.3.1. Microstructures of the Steels Tempered at 450 °C

**Figure 6:**
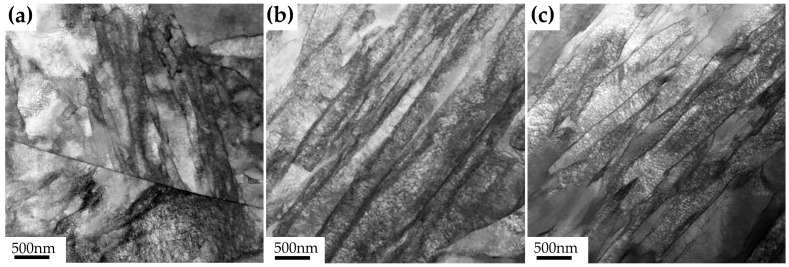
Bright field TEM images of the three steels with different Cu content tempered at 450 °C for 2 h: (**a**) 0Cu, (**b**) 1 wt.% Cu, and (**c**) 3 wt.% Cu.

Figure 6 presents the TEM images of the three steels with different Cu content tempered at 450 °C. It can be observed that the microstructures of the three samples are all composed of lath martensite. No reversed austenite or Cu-rich precipitate can be seen clearly, so we further used APT to characterize the three samples.

**Figure 7:**
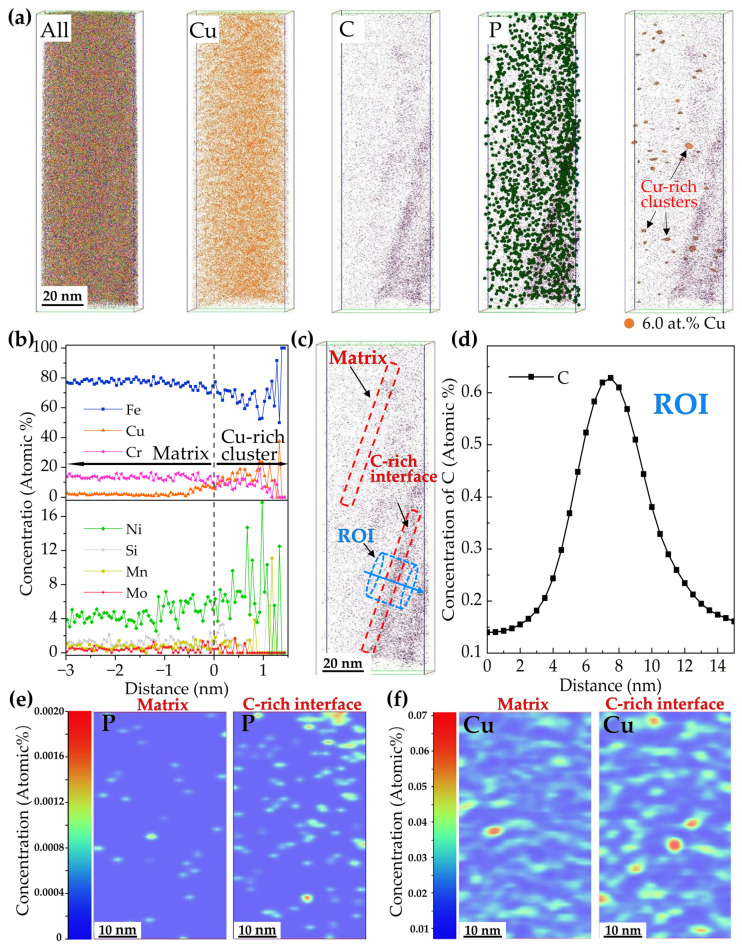
(**a**) The three dimensional (3-D) atomic probe maps and 3-D reconstructions based on isoconcentration surfaces of 6.0 at.% Cu in the 3Cu-steel tempered at 450 °C. (**b**) Proximity histograms of the Cu-rich clusters displayed in (**a**). (**c**) The 3-D atomic probe map of the C atoms and the region of interest (ROI), (**d**) a 1-D concentration profile of the ROI displayed by the light blue cylinder in (**c**). The 2-D concentration mappings of P (**e**) and Cu (**f**) taken from the “matrix” and “C-rich interface” regions are displayed by the red rectangles in (**c**).

Figure 7 shows the APT results of the 3Cu-steel tempered at 450 °C for 2 h. The Cu, C, and P atom mappings and the distributions of the Cu-rich clusters are displayed in Figure 7a. The corresponding proximity histogram obtained from the Cu-rich clusters is displayed in Figure 7b. It can be seen that the atoms located in the core of the Cu-rich cluster are mostly Fe, about 70 at.%. In addition, Ni and manganese (Mn) atoms are also enriched. C atoms can be found segregated at the interface. The peak C concentration at the interface reaches about 0.62 at.%, as shown in Figure 7d, which is about four times more than in the matrix. Therefore, the C-rich interface in this study is considered to be the martensitic lath interface, based on the theory reported by Lim, N.S. et al. [29]. Figure 7e,f show the 2-D concentration mappings of P and Cu atoms distributed in the matrix and the martensitic lath interface, respectively. From the mappings, it is easily observed that P and Cu have a higher concentration in the interface. This indicates that the existence of the C-rich interface is beneficial to the diffusion and redistribution of Cu and P.

#### 3.3.2. Microstructures of the Steels Tempered at 600 °C

**Figure 8:**
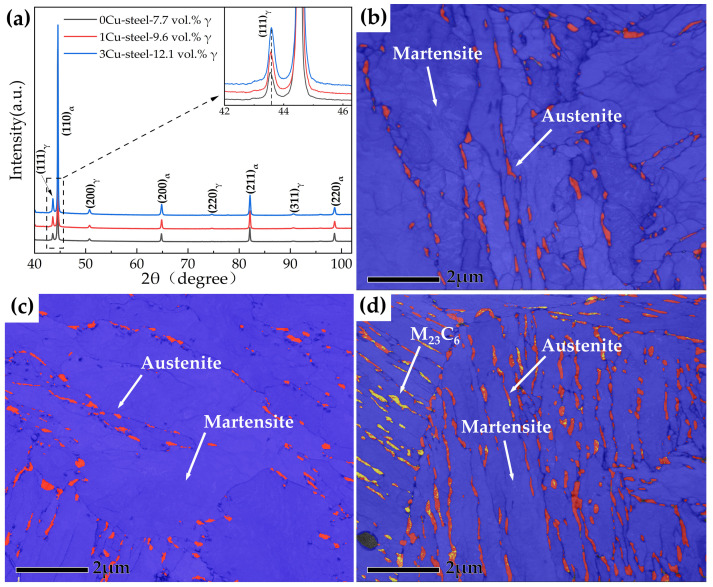
(**a**) XRD patterns of the three steels with different Cu content tempered at 600 °C; (**b**–**d**) depict the TKD band contrast (BC) and phase maps of 0Cu, 1Cu, and 3Cu steels tempered at 600 °C, respectively. The blue, red, and yellow colors signify the martensite, reversed austenite, and M_23_C_6_ phase, respectively.

The XRD patterns of the three steels with different Cu content are displayed in Figure 8a, and quantitative analyses of the volume fractions of the austenite phase in the 0Cu-steel, 1Cu-steel, and 3Cu-steel reveal they are 7.7%, 9.6%, and 12.1%, respectively. TKD phase maps of the three steels tempered at 600 °C are displayed in Figure 8b–d. It can be seen that a significant amount of reversed austenite (the red phase) can be observed in the three steels, which is mainly distributed at the prior austenite grain boundaries and the martensitic lath boundaries. The reversed austenite in 3Cu-steel is obviously more than that in 0Cu-steel and 1Cu-steel, and M_23_C_6_ (the yellow phase) can be clearly observed in 3Cu-steel, which mainly appears in the same position as the reversed austenite. This is consistent with the results reported by Niu, M.C. et al. where Cu accelerated austenite reversion in a maraging stainless steel [3].

**Figure 9:**
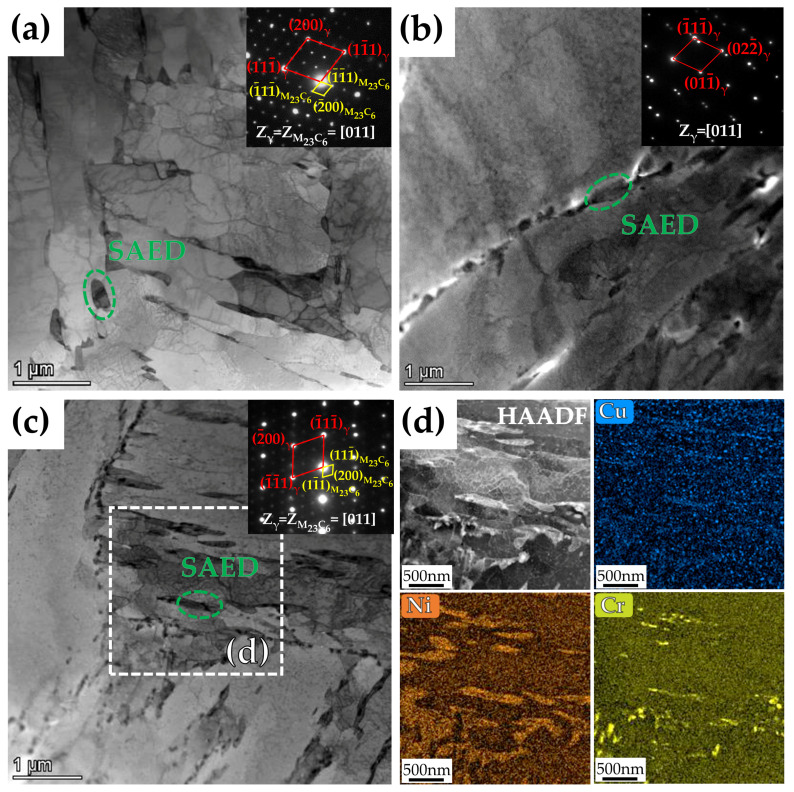
Bright field TEM images and the corresponding electron-diffraction analysis from the selected area of the three steels with different Cu content tempered at 600 °C for 2 h: (**a**) 0Cu-steel, (**b**) 1Cu-steel, and (**c**) 3Cu-steel. (**d**) The HAADF-STEM images and EDS mappings of Cu, Ni, and Cr corresponding to the white region in (**c**).

The detailed microstructures of the three steels with different Cu content tempered at 600 °C for 2 h were further analyzed by TEM, as shown in Figure 9. It can be detected that the reversed austenite is located at martensitic lath boundaries and grain boundaries, which are present in a granular or film form. In addition, Cr_23_C_6_ carbides were identified from selected area electron diffraction (SAED) and obeyed cube-on-cube orientation with the reversed austenite, i.e., [011]_Cr23C6_//[011]_γ_. Figure 9d shows the high-angle annular dark field scanning transmission electron microscope (HAADF-STEM) images and energy dispersive spectroscopy (EDS) mappings of Cu, Ni, and chromium (Cr) corresponding to the white region in (c), and Ni enrichment and Cr enrichment can be clearly observed in the reversed austenite. Ni is a reversed austenite forming element, and Cr enrichment is due to the presence of Cr_23_C_6_ carbides. Meanwhile, it can be observed from the STEM-EDS mapping of Cu that Cu-rich precipitates are dispersed in the matrix.

**Figure 10:**
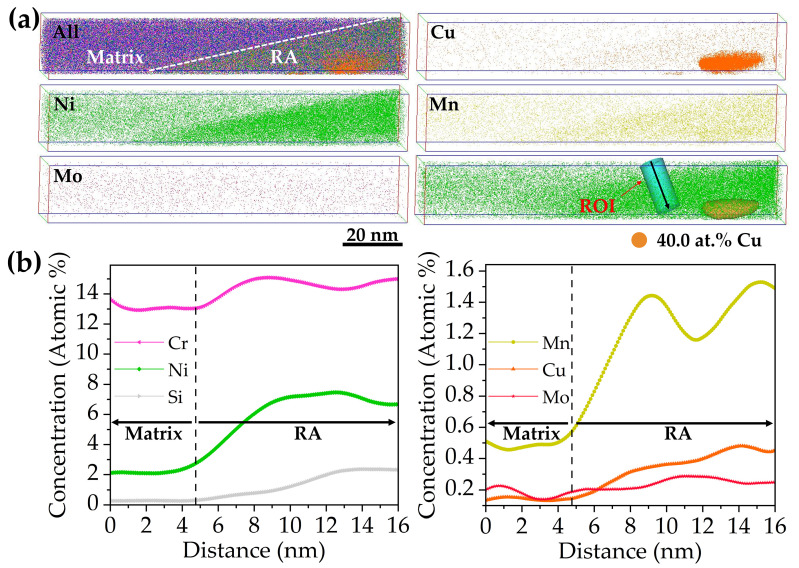
(**a**) The 3-D atomic probe maps of the 3Cu-steel tempered at 600 °C and the region of interest (ROI) and 3-D reconstruction based on isoconcentration surfaces of 40.0 at.% Cu in the Ni atom map. (**b**) The 1-D concentration profiles of the ROI displayed by the cylinder in (**a**). (For brevity, reversed austenite is denoted as RA.)

**Figure 11:**
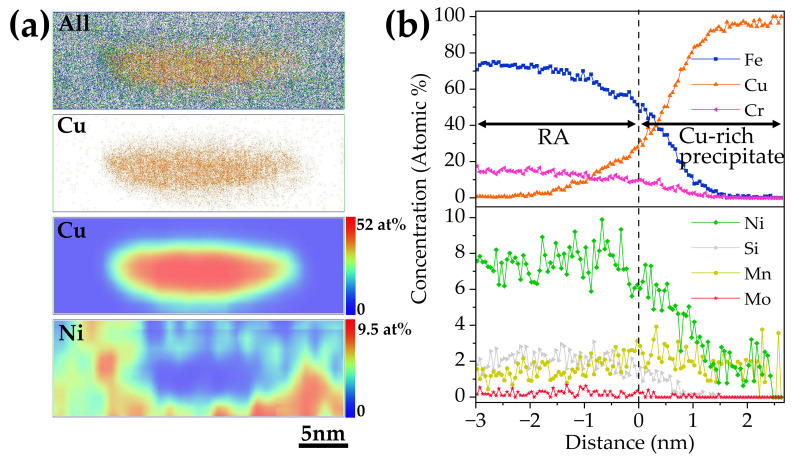
(**a**) The 3-D atomic mappings and 2-D concentration mappings of Cu and Ni in the Cu-rich precipitate displayed in Figure 10a. (**b**) The corresponding proximity histogram of the Cu-rich precipitate.

The APT results of 3Cu-steel tempered at 600 °C for 2 h are displayed in Figure 10. The segregation of Cu, Ni, and Mn atoms from the volume can be clearly observed. Based on the TEM results, the Ni-rich region can be identified as reversed austenite. Segregation of Mn atoms and Cu atoms formed as Cu-rich precipitates were found located in the reversed austenite. It can be observed from Figure 10b that the concentrations of Ni, silicon (Si), Mn, and Cu increased significantly from the martensite matrix to the reversed austenite. Figure 11a displays 3-D atomic probe mappings and 2-D concentration mappings of Cu and Ni atoms from one of the Cu-rich precipitates displayed in Figure 10a, and the corresponding proximity histogram is displayed in Figure 11b. From the histogram, it can be calculated that the concentration of Cu atoms reached nearly 100 at.% in the core of the precipitate. The concentration of Ni decreases toward the core of the Cu-rich precipitate. A shell structure of Ni can be clearly observed in Figure 11a. This is in agreement with the work of Wen, Y.R. et al. [30] where the Ni and Mn atoms segregated at the Cu precipitates, forming a core–shell structure in an age-hardened Fe-Cu-based multicomponent ferritic alloy.

### 3.4. Effect of Cu on the Formation of Reversed Austenite

From the experimental results shown in Table 2, it can be seen that both martensite and austenite phase transition temperatures are decreased with the increase in Cu content in the 0Cr13Ni4Mo low-carbon martensitic stainless steel, especially M_s_. The quantitative analysis of XRD results shows that Cu can significantly increase the amount of reversed austenite (Figure 8). These results indicate that Cu, as an austenite transformation element, plays a positive and significant role in promoting the formation and the stability of reversed austenite. APT results show that more Cu-rich clusters initially occur at the martensitic lath boundaries, which are good places for attracting alloy atoms. At a low tempering temperature, Cu atoms precipitate as nanoclusters, whose sizes are less than 5 nm. Cu composition at the core of the clusters is only ~14 at.%, and the other atoms are mainly Fe, Ni, and so on. When the tempering temperature increases, it accelerates atom diffusion. The Cu nanoclusters will grow with the increase in tempering temperature. Thus, the Cu atoms diffuse to the core of the precipitates gradually and, at the same time, other elements are expelled to the martensite matrix. The chemical compositions of the four types of microstructures are shown in Table 3, where the matrix and reversed austenite are the microstructures shown in Figure 10, the Cu-rich cluster is the microstructure shown in Figure 7, and the Cu-rich precipitate is the microstructure shown in Figure 11. It can be seen from the table that the concentration of Ni is 7.5 at.% in the reversed austenite, which is significantly higher than in the matrix. The concentration of Cu in Cu-rich precipitate is 78.3 at.%, which is dramatically higher than that of Cu-rich clusters (13.2 at.%); however, the concentration of Fe, Cr and Ni in Cu-rich precipitate is much lower than that of Cu-rich clusters. Additionally, Ni atoms are repelled to the interface between Cu-rich precipitates and the martensite matrix, which forms a Ni-rich shell (as shown in Figure 11). Ni atoms segregated at the interface between the Cu-rich precipitates and the martensite matrix can decrease interfacial energy according to previous studies [31,32,33]. Seko, A. et al. [31] found through first-principles calculation that the addition of Ni to a binary Fe-Cu alloy can reduce the activation barrier and thus reduce the critical nucleation work of a Cu cluster. Zhang, C. et al. [32] studied the effects of Mn and Ni on Cu precipitation in Fe-Cu alloys using the Langer–Schwartz simulation method based on the Cahn–Hilliard non-classical nucleation theory and indicate that both elements can reduce the nucleation work of body-centered cubic (bcc) Cu; the effect of Ni is better. In addition, Ni, as an austenite stabilizing element enriched in the vicinity of Cu-rich precipitate, will provide compositional conditions for the formation of reversed austenite. Therefore, Cu-rich precipitates are thought to be used as the site of the heterogeneous nucleation of reversed austenite, reducing the critical work for nucleation and inevitably promoting the formation and stability of reversed austenite.

### 3.5. Effect of Cu on Mechanical Properties

**Figure 12:**
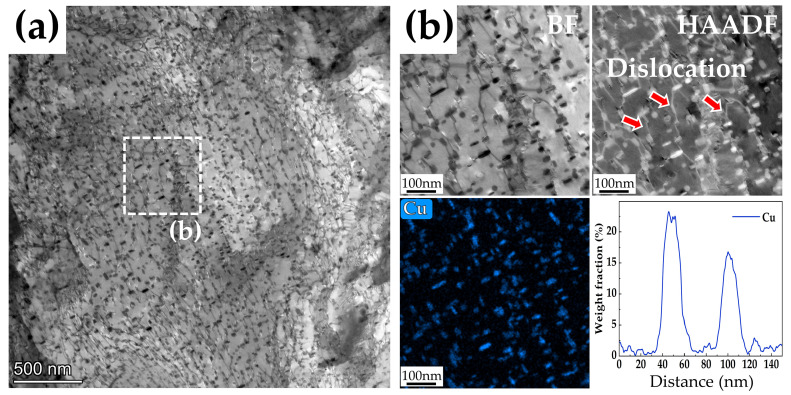
(**a**) Bright field TEM image of a 3Cu-steel tempered at 600 °C. (**b**) STEM images corresponding to the white region in (**a**).

It can be observed that the strength of the three steels gradually increased with the increase in Cu content, as shown in Figure 2. The reasons can be explained as follows. Due to the distribution of many Cu-rich precipitates of different size in the steels, dislocations can only pass through in the form of cutting or bypassing, which has a significant hindering effect on the movement of dislocations. It has been reported by previous researchers [3,34] that the solubility of Cu in martensite at 450 °C is very low (0.05 at.%), and the Cu atoms quickly form as coherent body-centered cubic (bcc) clusters with the martensite phase in the matrix during tempering. Interestingly, bcc clusters with diameters less than 5nm have extremely high strengthening effects in bcc ferritic or martensitic steels [35,36]. Therefore, when steel with 3 wt.% Cu addition is tempered at 450 °C, these small Cu-rich clusters of high number density (see Figure 7) precipitate and have a strong hindering effect on the movement of dislocations. This significant precipitate strengthening effect greatly increases the ultimate tensile strength and yield strength of 3Cu-steel to 1313 Mpa and 1131 Mpa, respectively. With the increase in tempering temperature, the size of Cu-rich clusters increases and the number density decreases, which weakens the precipitation strengthening effect. The pinning of dislocations by Cu-rich precipitates can be clearly observed from the HAADF-STEM images shown in Figure 12b. Therefore, the pinning effect of Cu-rich precipitates on dislocations can effectively improve the strength of the steel. Furthermore, the solid-solution strengthening caused by dissolved Cu in the matrix can also increase the tensile strength of the steels according to previous reports [3,19,28].

However, it should be noted that the 3Cu-steel presented obvious tempering brittleness after tempering at 450 °C (Figure 3). Cu promotes tempering brittleness for the following two reasons. Firstly, Figure 7 shows that more Cu precipitates were located on the martensitic lath boundary. This indicates that the existence of Cu-rich clusters weakens the binding force of the martensite lath boundary and becomes a potential crack source. Additionally, the Cu atoms can weaken the bonding force of Fe-Fe bonds on the lath boundary [37]. These findings are in good agreement with the results reported by previous researchers [38,39]. Secondly, due to the repulsion of Cu to Mo [40], the effect of Mo on improving tempering brittleness is restrained, which leads to the segregation of P on the martensitic lath boundary and increases the tempering brittleness of steel, as shown in Figure 7. Therefore, the impact toughness of the steel with 3 wt.% Cu addition was very poor after tempering at 450 °C, and the impact energy was only 7 J.

**Figure 13:**
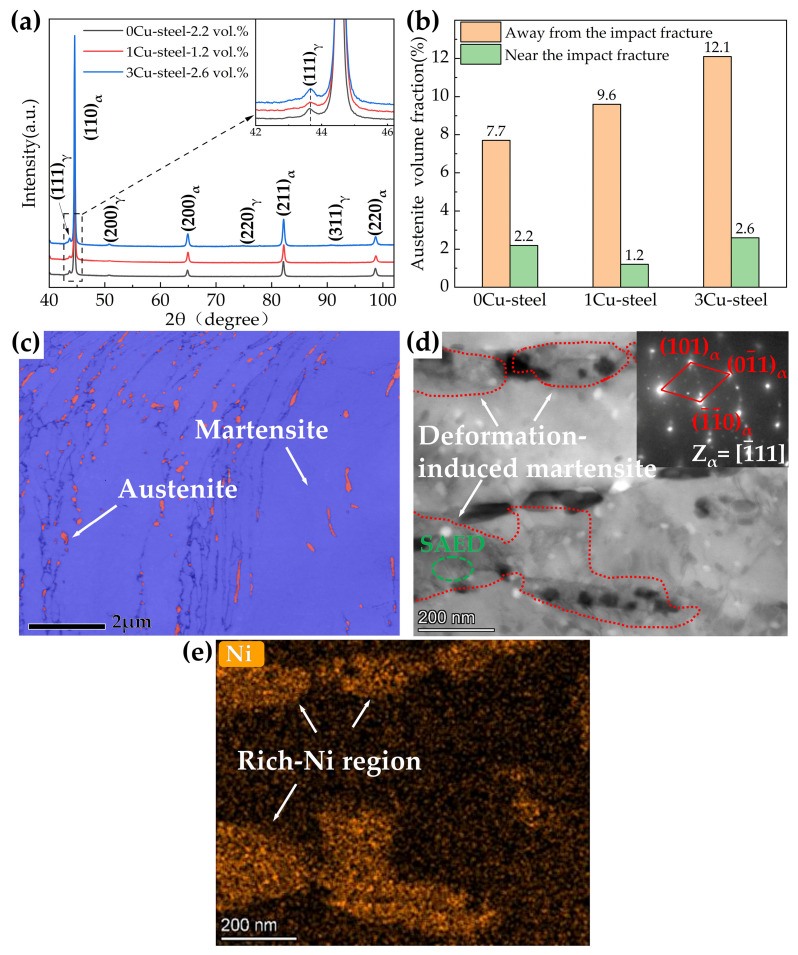
(**a**) XRD patterns of the three steels with different Cu content tempered at 600 °C near the impact fracture. (**b**) Volume fractions of reversed austenite near and away from the fracture. (**c**) The TKD band contrast (BC) and phase map of 3Cu-steels tempered at 600 °C near the impact fracture. (**d**) Bright field TEM image and the corresponding electron-diffraction analysis from the selected area of 3Cu-steels tempered at 600 °C. (**e**) EDS mapping of Ni for 3Cu-steels tempered at 600 °C.

The three steels all have good impact toughness after tempering at 600 °C, which is mainly because of the increased reversed austenite content during the tempering process, except for temper softening. Figure 13a shows the XRD patterns near the impact fracture, and quantitative analyses indicate that the volume fraction of the reversed austenite phase in 0Cu-steel, 1Cu-steel, and 3Cu-steel was 2.2%, 1.2%, and 2.6%, respectively. Comparison of reversed austenite content in the three steels before and after the impact experiments shown in Figure 13b indicates that the reversed austenite underwent phase transformation during impact deformation. This can be seen in Figure 13c,d in the remaining reversed austenite and new martensite. The result of EDS mapping suggests that the rich Ni region shown in Figure 13e was reversed austenite before impact deformation and was then transformed into new martensite (deformation-induced martensite) during impact deformation. The results of Figure 13 show that the reversed austenite was almost completely transformed into martensite after impact deformation. These findings reveal that reversed austenite improves impact toughness through the transformation-induced plasticity (TRIP) effect [27]. Additionally, the compressive stress caused by the volume expansion induced by the transformation of austenite to martensite can hinder the propagation of cracks [27]. It can be seen in Figure 13c that the remaining reversed austenite (red phase) in the steel is mainly smaller in volume, which is better for mechanical stability. It is also proposed in the literature [41] that untransformed fine reversed austenite increases the mean free path of cleavage cracks, which thereby improves the impact toughness of the steel. However, the room temperature impact test shows that, with increased Cu content, the impact toughness of steel does not increase but slightly decreases. This is due to the short tempering time (only 2 h) in our study, as Cu still existed at the grain boundary and martensite lath boundary, which had a negative effect on the strength of the interface. Therefore, the impact toughness of 3Cu-steel compared to 0Cu-steel and 1Cu-steel did not increase significantly.

This study breaks down the dilemma of increasing strength at the expense of toughness. 3Cu-steel tempered at 600 °C for 2 h not only has an ultimate tensile strength of 863 Mpa, but also has a ductility of 21% and an impact energy of 192 J, achieving suitable strength–ductility–toughness matching. On the premise of ensuring excellent impact toughness, tensile strength and yield strength are increased by 128 Mpa and 139 Mpa, respectively, compared to 0Cu-steel. This provides an economical and efficient heat treatment process for the processing and production of low-carbon martensitic stainless steels.

In addition, studies have shown that higher reversed austenite content decreases ductile–brittle transition temperature [16]; thus, the influence of Cu on ductile–brittle transition temperature is worthy of further study and discussion.

## 4. Conclusions

Based on a systematic study of the influence of Cu on the microstructure and mechanical properties of the 0Cr13Ni4Mo low-carbon martensitic stainless steel, the following conclusions can be put forward:

(i) The addition of Cu decreases martensite and austenite phase transition temperature, especially the starting temperature of martensitic transformation (M_s_). The volume fractions of reversed austenite increased with the increase in Cu content and were 7.7 vol.% for 0Cu-steel, 9.6 vol.% for 1Cu-steel, and 12.1 vol.% for 3Cu-steel tempered at 600 °C.
(ii) The samples tempered at 600 °C had a TRIP effect during impact deformation, and the content of reversed austenite in the three steels decreased significantly to only about 2 vol.%. In addition, deformation-induced martensite was observed in the microstructure near the impact fracture surface of 3Cu-steel.
(iii) APT results indicated that Cu-rich clusters initially formed with other alloying elements such as Fe and Ni and then grew to become precipitates through rejection of the alloying elements. The segregation of Ni atoms at the interface between the Cu-rich precipitates and the martensite matrix provided composition conditions for the formation of reversed austenite.
(iv) Cu precipitations increase tensile strength during tempering. However, the Cu precipitations also generate temper brittleness at a tempering temperature of 450 °C, resulting in the impact energy of the 3Cu-steel being only 7 J. The 3Cu-steel tempered at 600 °C for 2 h has an ultimate tensile strength of 863 Mpa, good ductility of 21%, and excellent impact energy of 192 J, achieving suitable strength–ductility–toughness matching.

## Figures and Tables

**Table 1:** Chemical compositions of the three steels studied (wt.%).

Steel	Cu	C	Si	Cr	Ni	Mn	Mo	P	S	Fe
0Cu-steel	0.01	0.051	0.43	12.87	4.06	0.68	0.47	0.005	0.0041	Bal.
1Cu-steel	1.17	0.056	0.51	13.06	4.15	0.67	0.49	0.005	0.0047	Bal.
3Cu-steel	2.82	0.056	0.51	13.03	4.12	0.66	0.48	0.005	0.0050	Bal.

**Table 2:** The phase transition points of the three steels.

Steel	A_s_ (°C)	A_f_ (°C)	M_s_ (°C)	M_f_ (°C)
0Cu-steel	687	790	255	100
1Cu-steel	674	770	241	91
3Cu-steel	674	763	190	85

**Table 3:** Chemical compositions in different microstructures of 3Cu-Steel (at.%).

Microstructure	Fe	Cr	Ni	Cu	Mn	Mo	Si
Matrix	82.2	13.0	2.0	0.1	0.5	0.2	0.9
Reversed austenite	72.8	14.4	7.5	1.0	1.4	0.2	2.0
Cu-rich cluster	69.3	7.8	6.2	13.2	0.9	0.2	1.3
Cu-rich precipitate	13.6	2.7	3.1	78.3	1.9	3.1	0.3

## Data Availability

Not applicable.
